# Exploratory Assessment of Muscle Thickness and Stiffness Around Arteriovenous Fistulas Using Shear Wave Elastography

**DOI:** 10.3390/jcm14248874

**Published:** 2025-12-15

**Authors:** Mohammed J. Alsaadi, Rana Alotaibi, Lina Fahmi Hammad, Maram AlOthman, Shagran Binkhamis, Abdulrahman M. Alfuraih

**Affiliations:** 1 Radiology and Medical Imaging Department, College of Applied Medical Sciences in Al-Kharj, Prince Sattam Bin Abdulaziz University, Al-Kharj 11942, Saudi Arabia; 2 Radiology and Medical Imaging Department, King Faisal Specialist Hospital & Research Centre, Riyadh 11433, Saudi Arabia; rmalotaibi@kfshrc.edu.sa (R.A.); malothman2@kfshrc.edu.sa (M.A.); sbinkhamis@kfshrc.edu.sa (S.B.); 3 Radiological Sciences Department, Applied Medical Science College, King Saud University, Riyadh 11433, Saudi Arabia; lhammad@ksu.edu.sa

**Keywords:** arteriovenous fistula, ultrasound, elastography, thickness, stiffness

## Abstract

**Background/Objectives**: Muscle thickness and stiffness near the arteriovenous fistula (AVF) site may influence vascular access function in hemodialysis patients. This exploratory study aimed primarily to describe differences in muscle thickness and stiffness between the AVF-bearing and contralateral limbs, and secondarily to examine whether these parameters were associated with AVF maturation. This study aimed to compare these muscle parameters between the AVF and contralateral sides and to evaluate their relationship with AVF maturation status. **Methods**: Thirty participants undergoing hemodialysis were included, comprising 22 with mature AVFs and 8 with non-mature AVFs. Ultrasound examinations measured muscle thickness and stiffness using Shear Wave Elastography (SWE) of the biceps brachii and brachioradialis muscles. Volume flow was recorded in the draining vein and feeding artery. Statistical analyses included paired comparisons, group comparisons, Pearson correlations, and multiple linear regression models. **Results**: Brachioradialis thickness was significantly reduced on the AVF side compared with the contralateral side (*p* = 0.013, Cohen’s d = –0.95), particularly in forearm (radiocephalic) fistulas (Wilcoxon *p* = 0.014, Cohen’s d = –0.95), indicating localized muscle atrophy. No significant side-to-side differences were found for biceps brachii thickness or for stiffness in either muscle (all *p* > 0.1). Comparisons between mature and non-mature AVF groups showed no significant differences in muscle parameters on the AVF side (all *p* > 0.4). **Conclusions**: AVF may lead to asymmetric muscle changes, especially a reduction in brachioradialis thickness, regardless of maturation status, which could affect forearm function. Nevertheless, muscle thickness and stiffness do not appear to predict AVF maturity or vascular access success directly.

## 1. Introduction

Arteriovenous fistula (AVF) creation is essential for enabling haemodialysis (HD) in patients with end-stage renal disease (ESRD) [1,2,3]. AVFs provide reliable vascular access by surgically connecting an artery and a vein, promoting blood flow that results in vein enlargement and wall thickening, making the vessel suitable for cannulation during HD procedures [2]. The process of fistula maturation involves structural remodelling of the vein, accompanied by a significant increase in blood flow, typically requiring a flow rate exceeding 500 mL/min for optimal maturation [2].

Patients often experience impaired upper-limb function, characterized by decreased mobility and discomfort due to significant vein dilation following AVF creation [3]. Such adverse outcomes are worsened when upper-extremity muscles have suboptimal structural qualities, particularly muscle stiffness and thickness [3,4].

The biomechanical properties of upper extremity musculature, particularly muscle thickness and stiffness, are crucial in supporting AVF maturation. These properties affect local blood flow and provide essential mechanical support for the remodelled vascular structure. Given the importance of successful AVF maturation for long-term dialysis treatment, it is notable that AVFs remain the preferred vascular access despite their high Failure-To-Mature (FTM) rate, which has been reported to range from 20% to 60% [5].

Failure of fistula maturation, often due to complex interactions between individual muscular anatomy and clinical factors, poses a significant clinical challenge. It is associated with increased morbidity, higher healthcare costs, and a considerable deterioration in patients’ quality of life [5,6,7]. Several interventions, including both regular and intermittent upper-limb exercises, have been shown to reduce arterial stiffness [8]. However, the effects of these exercises on upper-extremity musculoskeletal parameters and their subsequent impact on AVF outcomes remain insufficiently understood.

Although previous evidence has shown that arterial stiffness and muscle composition significantly influence AVF maturation, there remain significant gaps in understanding how the mechanical properties of upper limb muscles, particularly muscle thickness and stiffness, directly affect maturation [9,10]. Understanding the precise relationships between muscular mechanical properties and AVF maturation could guide targeted clinical interventions to increase maturation rates, thereby enhancing patient outcomes during chronic dialysis treatment.

Ultrasound has demonstrated strong accuracy and clinical value in assessing muscle status among hemodialysis patients. Quadricep thickness measured by ultrasound correlates closely with established sarcopenia criteria, confirming its reliability as a bedside biomarker of muscle mass [11]. Evidence from systematic reviews further supports ultrasound’s high diagnostic performance in detecting sarcopenia in CKD, validating its use for both muscle quantity and quality assessment [12]. Multi-modal ultrasound approaches—including thickness, echogenicity, and elastography—provide enhanced diagnostic precision, enabling comprehensive evaluation of muscle health in dialysis populations [13,14].

Shear Wave Elastography (SWE) provides an objective and sensitive measure of muscle stiffness in hemodialysis patients, enabling early detection of CKD-related musculoskeletal changes [15]. Evidence shows that SWE reliably identifies stiffness alterations linked to muscle dysfunction and neuropathy, offering advantages over conventional ultrasound [16]. Its reproducibility and ability to quantify subtle structural changes support its use as an accurate biomarker of muscle health. SWE represents a non-invasive and clinically meaningful tool for evaluating and monitoring muscle impairment in dialysis populations [17,18].

This exploratory study aimed primarily to describe differences in muscle thickness and shear wave stiffness between the AVF-bearing limb and the contralateral limb in patients undergoing hemodialysis. A secondary objective was to examine whether these muscle parameters are associated with AVF maturation status. Specifically, the research seeks to establish correlations between muscle structural features and the duration of AVF use, providing vital insights into the long-term musculoskeletal adaptations associated with chronic AVF use.

## 2. Materials and Methods

### 2.1. Study Design

A prospective, observational cohort study was carried out to assess and compare arm muscle thickness and stiffness in haemodialysis patients with arteriovenous fistulas (AVFs). All methods were carried out in accordance with relevant guidelines and regulations, including the Declaration of Helsinki and institutional requirements. Ethical approval was obtained from the Research Advisory Council at King Faisal Specialist Hospital and Research Centre (Approval No: RAC 2241383). Informed consent was obtained from all participants. Detailed study information, including objectives, procedures, potential risks, and benefits, was explained to participants. The study included 30 patients over 3 months. Inclusion criteria included individuals aged 18 years or older with a functioning AVF in use for at least 6 months. Exclusion criteria encompassed those with previous significant trauma, infections, or upper limb surgeries following fistula creation. Patients with recent upper-limb physiotherapy or structured exercise programmes targeting the fistula arm were excluded to avoid confounding muscle adaptations unrelated to AVF status.

### 2.2. Sample Size and Sampling Technique

An a priori sample size calculation was performed using SPSS software, assuming a moderate effect size (Cohen’s d = 0.5), a power of 0.80, and an alpha level of 0.05. To identify significant differences in muscle characteristics, at least 30 participants were required. Non-probability convenience sampling was employed based on participant availability. Data collection took place during routine haemodialysis sessions, with measurements of muscle stiffness (m/s), thickness (mm), and AVF maturation status. AVF maturation is defined by volume flow > 600 mL/min, draining vein diameter > 5 mm, and canulation depth < 1 cm. Measurements from the contralateral arm were also obtained for comparison.

### 2.3. Data Collection

Tools and procedures data were systematically collected using structured forms that captured demographic data (age, weight, height, BMI), patient history (including exercise habits), ultrasound elastography measurements (muscle stiffness and thickness), AVF maturation status, and overall arm diameter. AVF characteristics, including anatomical location, time since creation, type of anastomosis (end-to-side vs. side-to-side), and any previous surgical or endovascular interventions, were recorded when available. A high-resolution ultrasound system with shear wave elastography (SWE) capabilities (GE Logic E10) was employed, combining grayscale imaging for muscle thickness assessment with Colour Doppler for AVF evaluation, measuring feeding artery and draining vein flow velocity, draining vein diameter, and blood flow volume.

### 2.4. Measurement Protocol

**Figure 1:**
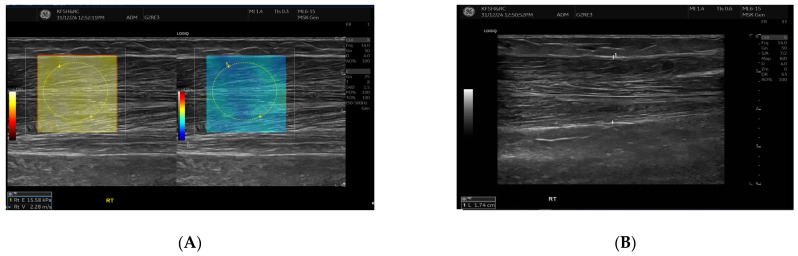
This figure shows the Ultrasound assessment of stiffness near the arteriovenous fistula site (**A**) and brachioradialis muscle thickness (**B**). Shear wave elastography images (**A**) obtained at the fistula side demonstrate quantitative stiffness mapping within a defined region of interest, with stiffness expressed in velocity in metres per second (m/s). B-mode image (**B**) showing brachioradialis muscle thickness measurement at the contralateral (**right**) arm.

**Figure 2:**
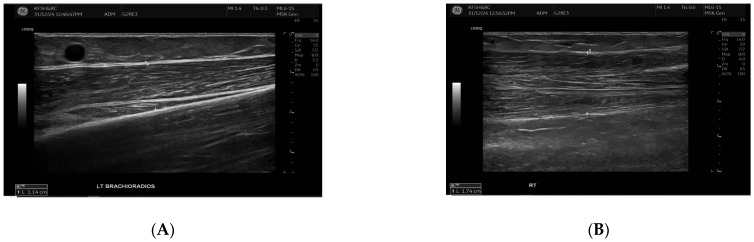
This figure demonstrates the comparison of brachioradialis muscle thickness between the fistula (**A**) and the contralateral arm (**B**). B-mode ultrasound images showing brachioradialis muscle thickness measurements. The **left** panel (**A**) (fistula side) demonstrates reduced muscle thickness (1.14 cm) compared with the contralateral arm (**right** panel (**B**), 1.74 cm). Measurements were obtained in the mid-muscle belly using a linear high-frequency transducer.

**Figure 3:**
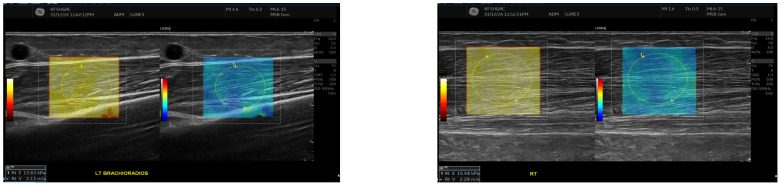
This figure shows the comparison of brachioradialis muscle stiffness between the fistula and the contralateral arm. Shear wave elastography images showing stiffness measurements of the brachioradialis muscle. The **left** panel (fistula side) demonstrates a mean elasticity (velocity 2.13 m/s), while the contralateral arm (**right** panel) shows slightly higher stiffness (velocity 2.28 m/s). Measurements were taken in the mid-muscle belly using a high-frequency linear transducer, with Colour maps indicating relative stiffness (warmer colours represent higher stiffness).

SWE measurements were acquired using a GE LOGIQ E10 (GE HealthCare, Chicago, IL, USA) system equipped with an ML 6-15 D linear transducer (frequency 6–15 MHz). Depth settings were standardized at 2.5–3.5 cm depending on muscle thickness. Each ROI was circular with a 5–7 mm diameter, placed centrally within the elastograms, avoiding fascia, vascular structures, and visible artefacts. Stiffness values were recorded in both kilopascals and metres/second, although statistical analyses were conducted using shear-wave velocity (m/s) for consistency. Frames with motion or colour dropout were discarded and reacquired. Muscle stiffness and thickness were evaluated in the biceps brachii and brachioradialis muscles, located proximal to the AVF site, Figure 1 and Figure 2. SWE imaging provided both Colour-coded elastograms and quantitative stiffness measurements (m/s; Figure 3). Participants were examined supine, with the shoulder in neutral rotation, the elbow extended to approximately 10–20°, and the forearm supinated. This minimized passive tension, which can influence shear-wave velocity. The transducer was positioned perpendicular to the muscle fibres, capturing measurements from the proximal, middle, and distal regions for comprehensive analysis. Measurements were taken before the haemodialysis to avoid intravascular volume shifts, with each parameter recorded three times and averaged for precision. Scans were performed by an experienced sonographer and verified by a radiologist, minimizing bias. A preliminary review of some patient records was conducted to refine data collection tools before the main data collection.

### 2.5. Data Analysis

Data were collected and initially cleaned using Excel spreadsheets. Statistical analyses were conducted using SPSS version 27. The Shapiro–Wilk test was used to assess normality. Normally distributed variables are presented as mean ± SD and compared between mature and non-mature AVF groups using independent-samples *t*-tests. Non-normally distributed variables are reported as median (IQR) and compared using the Mann–Whitney U test. Categorical variables were compared using χ^2^ or Fisher’s exact tests. Statistical significance was defined as *p* < 0.05.

Paired *t*-tests were used to compare muscle stiffness and thickness between the AVF and contralateral limbs within the same participants, both overall and stratified by AVF maturity. Independent *t*-tests were used to compare AVF-side muscle properties between mature and non-mature AVF groups. Because several variables did not meet the assumptions of normality or linearity, Spearman’s rank correlation coefficient (ρ) was used to evaluate associations between AVF-side muscle parameters (stiffness and thickness) and fistula characteristics (fistula age and draining vein volume flow). All statistical results are reported with corresponding test statistics, confidence intervals, and *p*-values. High-resolution visualizations (boxplots and scatter plots) were generated using seaborn and matplotlib.

## 3. Results

### 3.1. Participant Demographics and Clinical Characteristics

A total of 30 hemodialysis patients were included in the analysis, comprising 22 individuals with mature AVFs and 8 with non-mature AVFs. The baseline characteristics of both groups are presented in Table 1 and Table 2. There were no significant differences in age, height, or weight between groups (all *p* > 0.14). BMI, however, was significantly higher in the non-mature AVF group compared with the mature group (28.03 ± 4.13 vs. 23.96 ± 4.92 kg/m^2^; *p* = 0.037).

Fistula age demonstrated a non-significant trend toward being longer among patients with mature AVFs (median 5.41 years) than those with non-mature AVFs (median 2.62 years; *p* = 0.086). Hemodynamic parameters differed between groups: draining vein velocity and draining vein volume flow were markedly higher in the mature AVF group (median 136.98 cm/s vs. 55.56 cm/s and 1633.28 mL/min vs. 337.51 mL/min, respectively), with both variables showing statistically significant differences (*p* = 0.003 and *p* = 0.002, respectively). Feeding artery velocity and feeding artery volume flow did not differ significantly between groups (*p* = 0.507 and *p* = 0.070, respectively). Vein diameter was also comparable between groups (*p* = 0.192). Regarding comorbidities, hypertension was more prevalent in the non-mature group (62.5% vs. 45.5%), whereas diabetes mellitus was absent in this cohort. The baseline characteristics indicate that hemodynamic markers—particularly draining vein velocity and volume flow—were the primary differentiators between mature and non-mature AVFs. In contrast, patient demographic and anthropometric variables were largely similar.

### 3.2. Muscle Stiffness and Thickness Measurements

**Figure 4:**
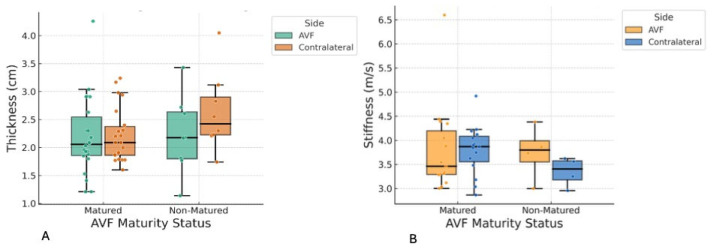
These figures show biceps brachii muscle thickness and stiffness in the arteriovenous fistula (AVF) and contralateral arms, stratified by AVF maturity. Box-and-whisker plots showing (**A**) muscle thickness (cm) and (**B**) shear wave velocity (m/s) of the biceps brachii in matured and non-matured AVFs compared with the contralateral arm. Data points represent individual measurements; boxes indicate interquartile range, horizontal lines represent medians, and whiskers show data range excluding outliers.

**Figure 5:**
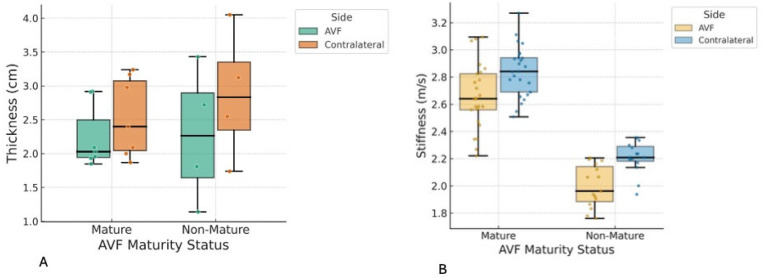
These figures showed the Brachioradialis muscle thickness and stiffness in arteriovenous fistula (AVF) and contralateral arms, stratified by AVF maturity. Box-and-whisker plots showing (**A**) muscle thickness (cm) and (**B**) shear wave velocity (m/s) of the brachioradialis in matured and non-matured AVFs compared with the contralateral arm. Each dot represents an individual measurement; boxes indicate the interquartile range, horizontal lines represent median values, and whiskers denote the data range excluding outliers.

Muscle stiffness and thickness of the biceps brachii and brachioradialis were assessed and compared according to AVF maturation status. Variables demonstrating normal distributions were reported as mean ± SD, whereas non-normally distributed parameters were summarized as median (IQR), consistent with the statistical approach used (Table 3 and Table 4; Figure 4 and Figure 5). Across both muscle groups, no statistically significant differences were observed between patients with mature and non-mature AVFs for any stiffness or thickness measure. Similarly, within-subject comparisons between the AVF-bearing and contralateral limbs revealed no significant asymmetry in stiffness or thickness for either muscle.

### 3.3. Side-to-Side Comparisons

Paired *t*-tests and Wilcoxon signed-rank tests were employed to compare muscle stiffness and thickness between the AVF and contralateral sides, both overall and stratified by maturity status. Effect sizes were quantified using Cohen’s d (Table 5 and Table 6). A significant reduction in brachioradialis thickness was observed on the AVF side overall (paired *t*-test: *p* = 0.013, Cohen’s d = −0.95; Wilcoxon: *p* = 0.014, Cohen’s d = −0.95), indicating a large effect. This difference approached significance in the mature subgroup (*p* = 0.055) but was not significant in the non-mature subgroup. No significant side-to-side differences were found for biceps brachii stiffness or thickness, or brachioradialis stiffness (all *p* > 0.1, with small to negligible effect sizes).

### 3.4. Maturity Group Comparisons

Unpaired *t*-tests and Mann–Whitney U tests were conducted to compare AVF-side stiffness and thickness between mature and non-mature groups (Table 7 and Table 8). No significant differences were identified for any parameter (all *p* > 0.4), with effect sizes ranging from negligible to medium.

### 3.5. Correlations with Fistula Age and Volume Flow

Spearman’s correlation coefficients were calculated to examine associations between AVF-side muscle stiffness/thickness and fistula age or draining vein volume flow (Table 9). No significant correlations were observed (all *p* > 0.2), suggesting that these muscle parameters are not strongly linked to fistula duration or hemodynamic flow.

### 3.6. Subgroup Analysis by Fistula Type

Fistulas were categorized as forearm (radiocephalic; n = 11, AVF-side thickness = 2.25 ± 0.67 cm) or upper arm (brachiocephalic, brachiobasilic, or graft; n = 19, AVF-side thickness = 2.18 ± 0.80 cm). No significant difference in AVF-side thickness was detected between these groups (t = 0.28, *p* = 0.780, Cohen’s d = 0.10). However, the side-to-side difference in brachioradialis thickness was significant in the forearm fistula subgroup (Wilcoxon *p* = 0.014, Cohen’s d = −0.95).

### 3.7. Multiple Linear Regression Models

**Figure 6:**
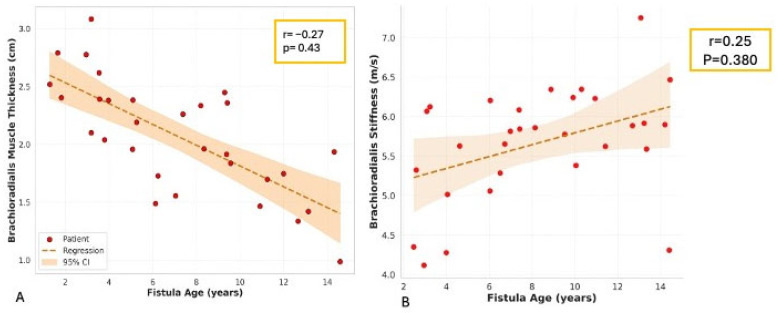
These figures showed the correlation of brachioradialis muscle thickness and stiffness with fistula age. Scatter plots with linear regression lines and 95% confidence intervals (shaded areas) showing the relationship between fistula age (years) and (**A**) muscle thickness (cm) and (**B**) shear wave velocity (m/s). Pearson correlation coefficients (r) and *p*-values are displayed on each plot.

Multiple linear regression analyses were performed to predict AVF-side muscle thickness using fistula age (years), maturity status (binary: 1 = mature), patient age, and BMI as independent variables (Table 10 and Table 11), Figure 6. Both models exhibited poor fit, with low R^2^ values and no significant predictors (all *p* > 0.2), implying that other factors may influence muscle changes.

## 4. Discussion

In this exploratory study, shear wave elastography (SWE) was applied to quantify the mechanical properties of the biceps brachii and brachioradialis muscles in hemodialysis patients with an arteriovenous fistula (AVF). The use of SWE allowed us to describe variations in muscle stiffness and thickness between limbs; however, these measurements should be interpreted as associative rather than causal, acknowledging that muscle characteristics may reflect pre-existing differences unrelated to AVF creation.

In this study, shear wave elastography (SWE) was utilized to quantify the mechanical properties of the biceps brachii and brachioradialis muscles in patients with end-stage renal disease receiving hemodialysis via arteriovenous fistula (AVF). The principal finding was a statistically significant reduction in brachioradialis muscle thickness on the AVF-bearing side, with no parallel changes in the biceps brachii, and no significant association between muscle parameters and AVF maturation, fistula age, or flow. These results indicate that AVF creation induces localized musculoskeletal adaptation, particularly in forearm muscles, with potential functional and clinical implications. The findings of this study should be interpreted as associative rather than causal. Given the cross-sectional design and the absence of pre-AVF baseline measurements, the observed differences in muscle thickness and stiffness cannot be attributed solely to the creation or maturation of the AVF. Muscle asymmetry may have been pre-existing, influenced by factors such as limb dominance, habitual physical activity patterns, occupational demands, or age-related sarcopenia. Therefore, our results should be considered hypothesis-generating, highlighting a potential relationship that warrants further validation in longitudinal studies with pre-procedural assessments.

The most notable finding of this study is the significant reduction in brachioradialis muscle thickness on the AVF side compared to the contralateral side, with a large effect size (Cohen’s d = −0.95), especially evident in patients with forearm (radiocephalic) fistulas. This asymmetry was not observed in the biceps brachii muscle, and no significant differences in muscle stiffness were observed between the two muscle groups. Additionally, AVF maturity status, fistula age, and draining vein volume flow did not correlate with these muscle parameters, and regression models did not identify significant predictors among the examined covariates, suggesting that muscle changes may be driven by local hemodynamic or mechanical factors rather than by gradual ageing.

Clinically, these results emphasize the potential for AVF creation, especially in the forearm, to cause localized muscle atrophy, which could weaken grip strength, impact daily functions, or even lead to fistula complications in hemodialysis patients. Nephrologists and vascular surgeons should routinely monitor forearm muscle health using non-invasive imaging techniques, such as ultrasound elastography, to detect early changes. Interventions such as targeted resistance training or physiotherapy may help reduce atrophy and enhance fistula maturation, while favouring upper arm fistula placement in at-risk patients (e.g., those with higher BMI or hypertension) could minimize musculoskeletal issues and support better long-term outcomes.

Shear wave elastography is increasingly recognized as a robust, non-invasive modality for evaluating muscle stiffness and quality, providing quantitative insight into tissue composition changes such as fibrosis and fat infiltration, and reflecting adaptation to chronic disease or vascular interventions [19,20]. Our findings of reduced brachioradialis thickness on the AVF side are consistent with the hypothesis of regional muscle atrophy or remodelling in response to altered hemodynamic and usage patterns following AVF creation. This pattern aligns with previous SWE studies reporting regional variability in muscle properties influenced by vascular and mechanical factors [21,22].

Notably, biceps brachii muscle stiffness and thickness did not differ significantly between the AVF and contralateral limbs. This may be attributable to anatomical differences and the location of the AVF site, as upper-arm AVFs are less likely to directly affect the biceps compared to how forearm AVFs affect the brachioradialis. Muscle stiffness, as measured by SWE, is highly site-dependent within the same muscle and between muscles with different vascular supply and functional demands [23,24].

Importantly, no significant associations were detected between muscle properties and AVF maturation, age, or flow parameters. Thomas et al. and Farrington et al. similarly found that vascular and functional adaptations post-AVF occur primarily in the initial months and are not reliably predicted by arterial or venous flow alone [24,25,26]. While some studies have associated macrovascular parameters (e.g., arterial diameter, cardiac function) with AVF outcomes [25,27,28], our results suggest that local musculoskeletal adaptation follows a distinct trajectory.

The absence of significant associations between muscle stiffness or thickness and patient age or gender contrasts with some prior SWE literature in non-dialysis populations, where age-related declines in muscle stiffness and function are consistently reported [26,27,29]. In our cohort, the mechanical and hemodynamic changes induced by AVF creation may have outweighed these systemic effects, highlighting the unique environment in dialysis patients.

The clinical relevance of these findings is considerable. Localized atrophy of the brachioradialis in the AVF limb could predispose patients to reduced forearm strength, grip, and function, potentially affecting quality of life and independence. Studies of limb function in hemodialysis patients have shown that musculoskeletal adaptation can translate into measurable impairment and impact daily activities [27,30,31,32,33]. Incorporating quantitative muscle assessment using SWE into routine clinical follow-up may facilitate early detection of such changes and enable timely intervention through rehabilitation or patient education.

This study is subject to several limitations. The relatively small sample size and single-centre design may restrict the generalizability of findings. The small size of the non-mature AVF group reflects the natural distribution of AVF maturation in our clinical population rather than data loss. While this limits statistical power and the ability to detect modest associations, it aligns with the study’s exploratory, hypothesis-generating intent. Notably, no participants were excluded, and group-specific sample sizes vary only because anatomical measurements were available for each muscle. SWE measurements can be operator-dependent, but all scans were performed by a single experienced sonographer following standardized protocols. Intra- and interobserver reproducibility for SWE measurements were not assessed, which limits the ability to generalize measurement reliability. Future work should include repeated measures across observers. Residual confounding by factors such as physical activity, comorbidities, and dialysis prescription cannot be excluded. Muscle morphology before AVF creation was unknown; thus, pre-existing asymmetry cannot be excluded. Future longitudinal studies with baseline ultrasound and elastography measurements are needed to determine whether observed changes truly reflect AVF-related remodelling.

Future studies should adopt longitudinal designs to determine the temporal evolution of muscle adaptation before and after AVF creation. Combining SWE with functional assessments such as grip strength, electromyography, or activity monitoring may clarify the clinical significance of muscle asymmetry. Larger, multicenter studies are needed to validate normative SWE values in hemodialysis populations. Additionally, interventional trials evaluating targeted physiotherapy or resistance training could determine whether modifying muscle stiffness or thickness improves AVF performance or patient functional outcomes.

## 5. Conclusions

In haemodialysis patients, the presence of an arteriovenous fistula (AVF) is associated with a localized reduction in brachioradialis muscle thickness and increased stiffness on the AVF-bearing limb, as quantified by shear wave elastography (SWE). These musculoskeletal alterations appear to be independent of AVF maturation status, blood flow parameters, or fistula duration, suggesting that they may represent an early and stable adaptive response to the haemodynamic and mechanical changes induced by AVF creation. In contrast, no comparable changes are observed in the biceps brachii muscle or in the contralateral limb, underscoring the site-specific nature of these adaptations. Routine quantitative muscle evaluation using SWE may facilitate early identification of patients at risk of functional decline, thereby enabling targeted, individualized rehabilitation strategies.

## Figures and Tables

**Table 1:** Descriptive Statistics for Continuous Participant Variables (Mean ± SD normally distributed) and Median (IQR) (non-normally distributed).

Variable	Mature AVF (n = 22)	Non-Mature AVF (n = 8)	*p*-Value (*t*-Test)
Age (years) ^†^	45.50 ± 14.91 (95% CI: 38.89–52.11)	46.50 ± 13.30 (95% CI: 35.38–57.62)	0.852
Height (cm) ^†^	155.66 ± 15.31 (95% CI: 148.87–162.45)	155.44 ± 8.89 (95% CI: 148.01–162.87)	0.968
Weight (kg) ^†^	58.67 ± 16.66 (95% CI: 51.29–66.06)	67.55 ± 8.06 (95% CI: 60.81–74.29)	0.143
BMI (kg/m^2^) ^†^	23.96 ± 4.92 (95% CI: 21.77–26.16)	28.03 ± 4.13 (95% CI: 24.58–31.49)	0.037
Fistula Age (years) ^‡^	5.41 ± 5.21 (3.17–7.64)	2.62 ± 1.51 (1.37–3.88)	0.086
Feeding Artery Velocity (cm/sec) ^†^	179.31 ± 93.13 (95% CI: 148.34–210.28)	155.47 ± 59.93 (95% CI: 105.37–205.58)	0.507
Feeding Artery Volume Flow (mL/min)	1332.99 ± 855.63 (95% CI: 963.20–1702.80)	725.15 ± 156.22 (95% CI: 590.40–859.90)	0.070
Vein Diameter (cm) ^‡^	1.04 ± 0.64 (95% CI: 0.77–1.31)	0.75 ± 0.27 (95% CI: 0.54–0.96)	0.192
Draining Vein Velocity (cm/sec) ^‡^	136.98 ± 69.86 (95% CI: 106.80–167.10)	55.56 ± 25.19 (95% CI: 36.90–74.20)	**0.003**
Draining Vein Volume Flow (mL/min) ^‡^	1633.28 ± 1217.71 (95% CI: 1124.20–2142.30)	337.51 ± 182.41 (95% CI: 181.10–493.90)	**0.002**

Note: ^†^ Normally distributed (Mean ± SD, 95% CI) and ^‡^ Non-normally distributed (Median (IQR)).

**Table 2:** Descriptive Statistics for Categorical Participant Variables [Counts (%)].

Variable	Mature AVF (n = 22)	Non-Mature AVF (n = 8)	*p*-Value (Chi-Square)
Gender	Female: 13 (59.1%), Male: 9 (40.9%)	Female: 5 (62.5%), Male: 3 (37.5%)	0.885
Diabetes Mellitus (DM)	No: 19 (86.4%), Yes: 3 (13.6%)	No: 8 (100.0%)	0.272
Cardiovascular Disease (CVD)	No: 22 (100.0%)	No: 8 (100.0%)	1.000
Stroke	No: 22 (100.0%)	No: 8 (100.0%)	1.000
Hypertension (HTN)	No: 12 (54.5%), Yes: 10 (45.5%)	No: 3 (37.5%), Yes: 5 (62.5%)	0.416
Smoker	No: 20 (90.9%), Yes: 2 (9.1%)	No: 7 (87.5%), Yes: 1 (12.5%)	0.792
Fistula Location	Left: 18 (81.8%), Right: 4 (18.2%)	Left: 8 (100.0%)	0.176

**Table 3:** Descriptive Statistics for Biceps Brachii Muscle ((Mean ± SD normally distributed) and Median (IQR) (non-normally distributed).

Parameter	Mature AVF (n = 15)	Non-Mature AVF (n = 4)	*p*-Value (*t*-Test)
AVF Stiffness (m/s) ^‡^	3.81 ± 0.92 (3.30–4.32)	3.75 ± 0.57 (2.84–4.66)	0.900
Contralateral Stiffness (m/s) ^†^	3.80 ± 0.52 (95% CI: 3.51–4.09)	3.35 ± 0.31 (95% CI: 2.86–3.84)	0.117
AVF Thickness (cm) ^†^	2.18 ± 0.80 (95% CI: 1.73–2.62)	2.18 ± 0.34 (95% CI: 1.64–2.73)	0.992
Contralateral Thickness (cm) ^†^	2.07 ± 0.37 (95% CI: 1.87–2.28)	2.39 ± 0.29 (95% CI: 1.92–2.86)	0.124

Note: ^†^ Normally distributed (Mean ± SD, 95% CI) and ^‡^ Non-normally distributed (Median (IQR)).

**Table 4:** Descriptive Statistics for Brachioradialis Muscle (Mean ± SD normally distributed) and Median (IQR) (non-normally distributed).

Parameter	Mature AVF (n = 7)	Non-Mature AVF (n = 4)	*p*-Value (*t*-Test)
AVF Stiffness (m/s) ^†^	2.63 ± 0.65 (95% CI: 2.03–3.23)	2.34 ± 0.32 (95% CI: 1.83–2.86)	0.433
Contralateral Stiffness (m/s) ^‡^	2.96 ± 0.68 (2.33–3.59)	2.35 ± 0.15 (2.10–2.59)	0.102
AVF Thickness (cm) ^†^	2.24 ± 0.47 (95% CI: 1.81–2.67)	2.28 ± 1.01 (95% CI: 0.67–3.88)	0.940
Contralateral Thickness (cm) ^†^	2.54 ± 0.58 (95% CI: 1.91–3.05)	2.86 ± 0.97 (95% CI: 1.32–4.41)	0.522

Note: ^†^ Normally distributed (Mean ± SD, 95% CI) and ^‡^ Non-normally distributed (Median (IQR)).

**Table 5:** Paired *t*-Test Results for AVF vs. Contralateral Side.

Muscle	Parameter	Group	t-Statistic	*p*-Value	Cohen’s d
Biceps Brachii	Stiffness	Overall	0.43	0.672	0.10
Biceps Brachii	Stiffness	Mature	0.03	0.977	0.01
Biceps Brachii	Stiffness	Non-Mature	2.37	0.099	0.79
Biceps Brachii	Thickness	Overall	0.26	0.799	0.06
Biceps Brachii	Thickness	Mature	0.58	0.574	0.15
Biceps Brachii	Thickness	Non-Mature	−0.91	0.429	−0.45
Brachioradialis	Stiffness	Overall	−0.85	0.416	−0.27
Brachioradialis	Stiffness	Mature	−0.86	0.422	−0.33
Brachioradialis	Stiffness	Non-Mature	−0.02	0.984	−0.01
Brachioradialis	Thickness	Overall	−3.01	0.013	−0.95
Brachioradialis	Thickness	Mature	−2.38	0.055	−0.90
Brachioradialis	Thickness	Non-Mature	−1.95	0.146	−0.97

**Table 6:** Wilcoxon Signed-Rank Test Results for AVF vs. Contralateral Side.

Muscle	Parameter	Statistic	*p*-Value	Cohen’s d
Biceps Brachii	Stiffness	91.5	0.922	0.10
Biceps Brachii	Thickness	81.5	0.595	0.06
Brachioradialis	Stiffness	25.0	0.520	−0.27
Brachioradialis	Thickness	6.0	0.014	−0.95

**Table 7:** Unpaired *t*-Test Results for Mature vs. Non-Mature AVF (AVF Side Only).

Muscle	Parameter	t-Statistic	*p*-Value	Cohen’s d
Biceps Brachii	Stiffness	0.13	0.900	0.07
Biceps Brachii	Thickness	−0.01	0.992	−0.01
Brachioradialis	Stiffness	0.82	0.433	0.50
Brachioradialis	Thickness	−0.08	0.940	−0.03

**Table 8:** Mann–Whitney U Test Results for Mature vs. Non-Mature AVF (AVF Side Only).

Muscle	Parameter	Statistic	*p*-Value	Cohen’s d
Biceps Brachii	Stiffness	30.0	1.000	0.07
Biceps Brachii	Thickness	28.5	0.920	−0.01
Brachioradialis	Stiffness	19.0	0.412	0.50
Brachioradialis	Thickness	16.0	0.788	−0.03

**Table 9:** Spearman’s Correlation Results (Overall for Each Muscle).

Muscle	Variables	Spearman’s ρ (r_s_)	*p*-Value
Biceps Brachii	Fistula Age & AVF Stiffness	0.04	0.869
Biceps Brachii	Drain Flow & AVF Stiffness	−0.19	0.443
Biceps Brachii	Fistula Age & AVF Thickness	−0.27	0.258
Biceps Brachii	Drain Flow & AVF Thickness	−0.28	0.239
Brachioradialis	Fistula Age & AVF Stiffness	0.25	0.467
Brachioradialis	Drain Flow & AVF Stiffness	0.12	0.735
Brachioradialis	Fistula Age & AVF Thickness	−0.27	0.431
Brachioradialis	Drain Flow & AVF Thickness	−0.02	0.944

**Table 10:** Regression Results for Biceps Brachii Thickness.

Predictor	Coefficient	SE	*t*-Statistic	*p*-Value	95% CI	
					Lower	Upper
Intercept	2.842	1.567	1.813	0.091	−0.50	6.18
Fistula Age (years)	−0.039	0.035	−1.110	0.286	−0.11	0.04
Maturity (binary)	0.026	0.553	0.047	0.963	−1.15	1.20
Patient Age	−0.010	0.013	−0.762	0.459	−0.04	0.02
BMI	−0.001	0.048	−0.017	0.987	−0.10	0.10

CI: confidence interval, SE: standard error.

**Table 11:** Regression Results for Brachioradialis Thickness.

Predictor	Coefficient	SE	*t*-Statistic	*p*-Value	95% CI	
					Lower	Upper
Intercept	2.309	2.293	1.007	0.353	−3.50	8.11
Fistula Age (years)	−0.075	0.127	−0.592	0.576	−0.39	0.24
Maturity (binary)	0.212	0.498	0.426	0.685	−1.00	1.43
Patient Age	−0.013	0.025	−0.513	0.627	−0.08	0.05
BMI	0.025	0.063	0.398	0.704	−0.13	0.18

CI: confidence interval, SE: standard error.

## Data Availability

The data presented in this study are available within the article.

## Abbreviations

The following abbreviations are used in this manuscript:

AVF	Arteriovenous Fistula
US	Ultrasound
SWE	Shear Wave Elastography
HD	Haemodialysis
ESRD	End-Stage Renal Disease
FTM	Failure-To-Mature
GE	General Electric
